# Editorial: Defining the role of Artificial Intelligence (AI) in the food sector and its applications

**DOI:** 10.3389/fnut.2025.1596699

**Published:** 2025-04-08

**Authors:** Tarun Belwal, Rui Huang, Hefei Zhao

**Affiliations:** ^1^Department of Horticultural Science, Texas A&M University, College Station, TX, United States; ^2^Department of Food Science and Human Nutrition, University of Illinois Urbana-Champaign, Urbana, IL, United States; ^3^Department of Food Science and Technology, University of California, Davis, Davis, CA, United States

**Keywords:** food security, data integration algorithm, food waste management, agriculture sustainability, nutrition, crop management and monitoring, decision making, machine learning

The integration of Artificial Intelligence (AI) into the food sector is transforming how we approach agriculture, food safety, waste management, and nutrition. As AI continues to evolve, it is revolutionizing the way food systems operate by improving efficiency, reducing waste, and enhancing food quality. This Research Topic explores the growing role of AI in the food sector through a diverse collection of articles that highlight its potential applications, challenges, and future directions. In this editorial, we summarize the findings from several groundbreaking studies that reflect AI's ability to impact various aspects of food production, management, and sustainability.

## AI in agriculture: enhancing crop disease diagnosis and yield prediction

One of the most promising applications of AI in the food sector lies in its ability to improve agricultural practices. In the study by Alhammad et al., deep learning and explainable AI were employed for the classification of potato leaf diseases. By employing advanced machine learning techniques, the authors showed how AI could identify and classify diseases in potato crops, offering a precise and scalable solution to disease detection. This method significantly enhances early intervention and crop protection, improving overall agricultural productivity. Similarly, Mayo et al. utilized deep learning models to detect maize diseases, such as maize streak virus and maize lethal necrosis, in Tanzania. Their findings demonstrate how AI-driven diagnostic tools can revolutionize crop disease management, offering faster and more accurate identification than traditional methods. These AI systems not only detect diseases early but can also predict their spread, contributing to more effective and sustainable crop management. The work by Taniguchi et al. further exemplifies AI's role in improving agricultural productivity. Their study focused on phenology analysis using UAVs (unmanned aerial vehicles) in a MAGIC rice population, allowing for trait prediction across different transplanting protocols. By combining drone technology with AI, the researchers were able to monitor rice growth and predict crop outcomes with greater accuracy, a critical factor in enhancing yield prediction and resource allocation in agriculture.

## AI in food waste management: addressing sustainability challenges

Food waste remains a critical issue, with significant environmental and economic implications. Clark et al. explored the potential of AI-driven food waste management strategies in the hospitality industry, proposing that these techniques could be applied to household settings. Their study suggests that AI could streamline waste management processes, leading to reduced food wastage and improved sustainability in both commercial and household environments. By utilizing AI to predict consumption patterns, food waste could be minimized, and resources could be better allocated.

## AI for gender-inclusive innovations in food systems

AI's role extends beyond technological advancements; it also has the potential to drive inclusive and responsible innovation. Ozor et al. highlighted the importance of gender-inclusive AI innovations in enhancing agriculture and food systems in Africa. Their study, which examines insights from the AI4AFS network, emphasizes how AI can empower marginalized communities, particularly women in agricultural sectors, by providing them with better access to data and resources that improve food security and agricultural sustainability.

## AI in food recognition and nutritional analysis

The recognition and analysis of food images have emerged as key areas for AI development, facilitating the digitalization of food systems. Dai explored a deep learning-based refrigerator food recognition system that can identify and categorize various food items, helping consumers manage their food more effectively and reduce waste. This system utilizes AI to automatically detect food items, analyze their condition, and offer suggestions for usage or disposal, making it a powerful tool for smart kitchens. Similarly, Wang et al. introduced a novel Swin Transformer approach to nutritional composition analysis in food images. Their work enables the accurate assessment of nutritional information by analyzing food images, thus providing consumers with valuable insights into their dietary choices without the need for manual input. This AI-driven method can help promote healthier eating habits and assist in the personalized nutrition field.

## AI in enhancing food systems with data integration

AI's ability to integrate diverse data sources is another valuable asset in the food sector. Kierdorf et al. examined how image time series data could be used to predict cauliflower harvest readiness. By combining multiple data streams, including visual and temporal information, their AI model was able to accurately predict the optimal harvest time, ensuring better food quality and reduced waste. In another notable study, Kyalo et al. developed a decision support system using convolutional neural networks (CNN) that integrates image and numerical data to improve the farming of edible crickets as a sustainable food source. Their AI-driven system is designed to optimize farming conditions and predict cricket growth, demonstrating AI's potential to enhance the sustainability of alternative protein sources. Ali presents a Bayesian model designed to optimize wheat tilling practices in response to climatic challenges and sustainability concerns. The model integrates weather data, soil conditions, and crop growth patterns to provide actionable insights for farmers, helping them make informed decisions on when and how to till the soil for maximum yield. By addressing both environmental and sustainability aspects, the research offers a robust AI-driven approach to improve wheat farming, which is crucial for global food security. The study underscores the importance of leveraging AI to adapt traditional agricultural practices to the challenges posed by climate change.

## AI in food environment and market research

AI is not limited to food production and waste management; it also plays a critical role in understanding and improving the food environment. Etheredge et al. employed large language models (LLMs) to enhance food environment data by analyzing food store names and attributes. This application highlights AI's capability to mine vast amounts of unstructured data, leading to better insights into food availability, consumer behavior, and market trends. Furthermore, Ogrinc et al. applied AI for food named-entity recognition and linking, leveraging ChatGPT in zero-shot evaluations. Their study underscores how AI-powered tools can automatically recognize and link food-related entities, facilitating the creation of more efficient food databases and contributing to better decision-making in the food industry.

## The future of AI in the food sector

The contributions to this Research Topic illustrate the transformative potential of AI in the food sector. From disease detection in crops to the optimization of food recognition systems, AI is paving the way for smarter, more sustainable food systems. As AI technologies continue to advance, they will undoubtedly play an even more prominent role in tackling global challenges such as food security, waste reduction, and sustainable agriculture. The studies presented here show the vast range of applications AI can have across various stages of the food supply chain, from production to consumption. With continued innovation and the responsible integration of AI, the future of the food sector holds the promise of more efficient, equitable, and sustainable food systems worldwide.

